# Inaugural Address before the Fulton County Medical Society

**Published:** 1909-02

**Authors:** Cyrus W. Strickler

**Affiliations:** Atlanta, Ga.


### Journal-Record of Medicine

Successor to Atlanta Medical and Surgical Journal, Established 1855, and Southern Medical Record, Established 1870.

**OWNED BY THE ATLANTA MEDICAL JOURNAL CO.**

**Published Monthly.**

*Official Organ Fulton County Medical Society, State Examining Board, Presbyterian Hospital, Etc.*

EDGAR G. BALLENGER, M. D., Editor.
BERNARD WOLFF, M. D., Supervising Editor.
A. W. STIRLING, M. D., C. M., D. P. H., J. S. HURT, B. Ph., M, D,
GEO. M. NILES, M. D., Associate Editors. E. W. ALLEN, Business Manager.

COLLABORATORS

DR. W. F. WESTMORLAND, General Surgery.
F. W. McRAE, M. D., Abdominal Surgery.
H. F. HARRIS, M. D., Pathology and Bacteriology.
E. B. BLOCK, M. D., Diseases of the Nervous System.
MICHEL HOKE, M. D., Orthopedic Surgery.
CYRUS W. STRICKLER, M. D., Legal Medicine and Medical Legislation.
E. C. DAVIS, A. B., M. D., Obstetrics.
E. G. JONES, A. B., M. D., Gynecology.
R. T. DORSEY, Jr., B. S. M. D., Medicine.
L. M. GAINES, A. B., M. D., Internal Medicine.
J. N. LeCONTE, M. D., Diseases of the Stomach and Intestines
L. B. CLARKE, M. D., Pediatrics.
EDGAR PAULIN, M. D., Opsonic Medicine.
R. R. DALY, M. D., Medical Society.
A. W. STIRLING, M. D., Etc., Diseases of the Eye, Ear, Nose and Throat.
BERNARD WOLFF, M. D., Diseases of the Skin.
E. G. BALLENGER, M. D., Diseases of the Genito Urinary Organs.

VOL. XI. FEBRUARY, 1909. No. 11

### INAUGURAL ADDRESS BEFORE THE FULTON COUNTY MEDICAL SOCIETY.

BY CYRUS W. STRICKER, M. D., ATLANTA, GA.

As a medical student I attended the meetings of this society. Since becoming a member I have attended with a fair degree of regularity. During these years I have spent many pleasant hours in your midst. I have learned much, not only from the dear departed, but from those present. I love this society and have a keen interest in the welfare. Therefore, knowing this, you may better appreciate my feelings, when I learned you had honored me by making me your president for the year 1909. Especially am I grateful to you for this distinction, when I look around me and see so many more worthy and who would have graced the chair with signal ability. I thank you for your kindness and confidence, and assure you that with your generous and able assistance, I will lend my best efforts to make this the best year in our society's history, not for any praise that might come to me or any other individual member, but for the glory and common good of us all. I, therefore, ask each one of you to attend regularly and let us meet together with a unity of purpose, and in this way make each meeting profitable and pleasant to all.

This society under the leadership of our beloved and distinguished Scotchman, has made marked progress and I will endeavor to follow in his footsteps by advocating many of the measures noted by him in his annual address.

I wish to call your attention to the fact that the future success of this society depends upon its members. Unless you attend its meetings regularly, read papers, enter into the discussions and discharge faithfully all duties that may be imposed upon you, I see a failure in sight. I urge you to read papers, discuss papers and present cases, specimens and apparatus. I believe it would be a step in advance if this society would purchase suitable instruments and apparatus for the proper demonstration of cases.

I again call your attention to the great value of a library, and now that the opportunity for securing one is again offered, let each member contribute his share and help establish a library of which we can justly feel proud, and by using it, realize its value. It is needless for me to tell you that without it we will drift away and lose interest in scientific medicine and our ignorance shall become more appalling as the years go by. It is unnecessary and almost impossible for any one now to own all the literature necessary to keep him abreast of the times and stimulate in him the desire to make some advance in scientific medicine.

I suggest that the board of censors or a special committee be appointed and representing this society, call upon the editors of the various daily papers and insist that the names of its members be omitted when giving information regarding the illness of our various citizens. If our wish is not respected, certain demands might be made. It is high time that such a representative body of men take some steps to so unite in all their just desires, that their slightest wish would receive at least attention and all right and reasonable demands perfect respect. The high standard and dignity of our forefathers is sadly lacking in most of us at the present day. May we make honest efforts to stop this decline and endeavor to emulate the physician of the old school, who was loved by many, possibly disliked by a few(?) but respected by all.

I have heard constant complaint of the miserable telephone service given members of this society. Considering the amount paid in yearly by us and caused by us to be paid in, I think we have a right to demand accurate and reasonably rapid service. If it is your wish, I will appoint a committee to look after our interest in this matter.

I have been requested to call especial attention to the importance of keeping vital statistics and of using proper prophylactic measures to prevent ophthalmia neonatorum. It is only right to say, that neglect, however slight, in the latter is unpardonable and we should use our most earnest efforts to prevent this calamity following in the wake of the social evil.

It should be a matter of great concern, not only to the physician, but to the laity, that articles necessary for public education should regularly appear in the daily papers. Our people will soon learn to appreciate our interest in their well being by heeding the advice given. I believe a special committee should be appointed to look after this matter.

The true physician never withholds aid from the needy sufferer, neither does he practice his profession for even dollars, not gloat over the gold that may fill his pockets. He has a higher purpose, nevertheless, we have to live and meet our obligations. We must do our duty to those dependent upon us. Therefore it is time for us to make proper efforts to control the highwaymen who hold us up and take what we alone can give. A central collector, I believe, is the cure for this practice, and the sooner we unite on this or some other plan, the better it will be, not only for our protection, but more so for him who is worthy, and in need. Your former president suggested and urged this.

The recent agitation in regard to the automobile ordinance gave promise of working many hardships upon our members who use machines, but our thanks are due Dr. L. C. Fisher for taking this matter up and securing for us the liberty of driving as the urgency of our work demands. Chief Jennings has instructed his men to show doctors this courtesy and to only take action in cases of recklessness. Let us see to it that this privilege is not abused. In a short time some sign will be selected to designate our machines.

I have sent each member on our roll a communication requesting him to read one or more papers during the year. The program will be made out next week and it will be necessary for all titles to be in by that time. Kindly attend to this matter at your earliest opportunity.

The charlatan still resides and fattens in our midst. I urge that some effort be made to rid ourselves of, or curtail the operation of this blot on the page of medical history. This burglar for the unsuspecting and the innocent. If there is one really great service we could render the citizens of this city and state, it would be to forever banish this performer of miracles.

Many don't understand why this worker of wonders is not as honest and capable as he who guarantees nothing, promises little, but is ever honest and faithful in his efforts. I believe much can be done and I will urge the legislative committee to take this matter up and promise them my support.

I have thought it wise to utilize the time at our disposal in discussing plans for future welfare of our society. However, if any of the members desire the usual dinner speeches, their wish should be granted.

May we now bury all past grievances and have generated in our hearts a kindlier feeling and greater love each for the other, and put aside all that tends to disrupt or contaminate the whole. May there be no envy or strife in our midst, but an honest joy for him who succeeds. A helping hand and words of comfort and cheer for him who fails.

